# Evolutionary Dynamics of Intratumor Heterogeneity

**DOI:** 10.1371/journal.pone.0017866

**Published:** 2011-03-30

**Authors:** Yoh Iwasa, Franziska Michor

**Affiliations:** 1 Department of Biology, Faculty of Sciences, Kyushu University, Fukuoka, Japan; 2 Department of Biostatistics and Computational Biology, Dana-Farber Cancer Institute, and Department of Biostatistics, Harvard School of Public Health, Boston, Massachusetts, United States of America; University of Oxford, United Kingdom

## Abstract

Intraneoplastic diversity in human tumors is a widespread phenomenon of critical importance for tumor progression and the response to therapeutic intervention. Insights into the evolutionary events that control tumor heterogeneity would be a major breakthrough in our comprehension of cancer development and could lead to more effective prevention methods and therapies. In this paper, we design an evolutionary mathematical framework to study the dynamics of heterogeneity over time. We consider specific situations arising during tumorigenesis, such as the emergence of positively selected mutations (“drivers”) and the accumulation of neutral variation (“passengers”). We perform exact computer simulations of the emergence of diverse tumor cell clones over time, and derive analytical estimates for the extent of heterogeneity within a population of cancer cells. Our methods contribute to a quantitative understanding of tumor heterogeneity and the impact of heritable alterations on this tumor trait.

## Introduction

Human tumors originate from normal cells that accumulate genetic and epigenetic changes. The types and numbers of changes necessary for malignant transformation differ between tumor types, but a common feature among most types is variability in both genotype and phenotype among the cancer cells within a single tumor [1]–[3]. These cells can be distinguished by characteristics such as size, cellular morphology, and antigen expression as well as in behaviors like cell turnover, motility, cell-cell interactions, response to treatment, and angiogenic, immunogenic, and metastatic ability [4], [5]. This tumor diversity has profound clinical implications for disease progression, diagnosis, therapeutic responses, and the choice of optimal treatments. Since biopsies used for diagnostic purposes sample only a small region of a tumor, they might not be representative of the totality of a diverse cancer cell population; hence treatment choices based upon such diagnostic samples might not inhibit all tumor cells and thereby lead to residual disease in many patients. Similarly, the use of biomarkers may lead to inappropriate conclusions about the type, stage, and prognosis of a tumor. The therapeutic response of tumors also depends on the composition of the cell population: experimental tumors composed of multiple clones display different sensitivity to cytotoxic drugs as compared to monoclonal tumors, since clonal interactions can either potentiate or inhibit therapeutic efficacy [6]. Therefore, intratumor heterogeneity adds an additional level of complexity to the study of cancer development and poses challenges for the development of successful therapies.

Determining the evolutionary events that control tumor heterogeneity would increase our understanding of cancer development and could lead to more effective prevention methods and therapies. In this paper, we design a mathematical model of tumor heterogeneity and investigate the evolutionary dynamics of this tumor trait. The present work contributes to the study of tumor diversity using computational modeling [7]–[14]. Diversity and the accumulation of mutations have also been the subject of many research efforts in evolutionary theory [15]–[27]. The models presented in the population genetics literature generally consider cells undergoing sexual reproduction and situations with weak selection. However, the dynamics of selective sweeps of advantageous mutations differ between asexually and sexually reproducing populations, and the types of modeling approaches used to examine scenarios with weak selection cannot be applied to situations with strong selection such as those arising during cancer evolution. Hence a novel mathematical framework is necessary to investigate the extent of heterogeneity of an asexually evolving population accumulating alterations with large fitness effects over time. The model presented in this paper serves as a toy model to investigate the dynamics of tumor heterogeneity and the impact of heritable alterations on diversity. We consider an idealized mathematical model in which cells proliferate according to a stochastic process in which they maintain a strictly constant population size. The assumption of a constant population size may describe pre-malignant tissues in which genetic and epigenetic alterations lead to diversity before more aggressive clones arise. Additionally, such a model applies to cancer cell populations which have reached a carrying capacity due to resource limitation, the lack of an appropriate phenotype conferred by specific mutations, or other restrictions to continued growth such as the absence of angiogenesis, presence of a strong immune response, or tissue and compartment boundaries. Although the tumor cells may be able to continue to expand exponentially once those barriers are removed, for the duration of time until the evolution of a more aggressive phenotype, the assumption of a constant population size may apply. Therefore, we here analyze a model restricting the number of cells to a constant value over time.

## The Model

**Figure 1:**
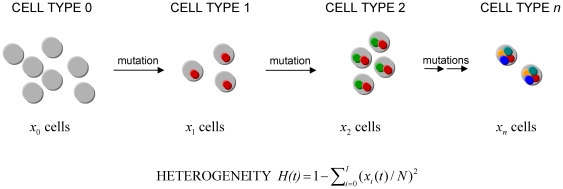
A mathematical model of tumor heterogeneity. We consider a population of *N* tumor stem cells which accumulate mutations. Each time a mutational event occurs, a new cell type is created. Cell types are enumerated *i* = 0, 1, …, *I*. The extent of heterogeneity at time *t* is measured by Simpson's index shown below, which incorporates the number of cells of each type at time *t*, 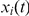, and the total number of cells, *N*.

Consider a population of *N* cancer cells following a stochastic process [28]: at each time step, a cell is chosen for replication proportional to fitness (i.e. growth rate), and its offspring replaces another randomly chosen cell. The population size remains strictly constant during the period of observation; the population size can, however, increase in response to the accumulation of specific (epi)genetic changes. During each cell division, a new genetic or epigenetic alteration may emerge in one of the two daughter cells. We consider two types of alterations: those that do not alter the growth or death rate of the cell (neutral or “passenger” changes), and those that confer a fitness advantage to the cell. The latter mutations may be “driver” alterations that contribute to tumor progression and malignancy. Deleterious mutations, i.e. those that decrease cellular fitness, are not considered since they will likely be lost from the population. Neutral and advantageous changes arise at rates *u* and *v* per cell division, respectively. Each time a mutational event occurs, a novel cell type arises (Fig. 1). This assumption is known as the infinite alleles model in population genetics [29]. Denote those cell types that do not harbor an advantageous mutation as type A cells (including all cell types carrying different numbers of neutral mutations), and those cells that harbor a specific advantageous mutation as type B cells. The growth rates of type A and B cells are given by *R* and *R+S*, respectively, while both their death rates are given by *D*. Note that we assume the fitness effect of the advantageous alteration to act on the growth rather than the death rate; alternative assumptions are possible. Denote by *H*(*t*) the probability at time *t* that a randomly chosen pair of cells is genetically distinct; this quantity is called tumor heterogeneity.

In tumors that follow the stem cell hypothesis – i.e. the idea that only a small subset of tumor cells possesses unlimited self-renewing abilities [30], only cancer stem cells can accumulate variability that will persist in the population. All alterations arising in transit-amplifying or terminally differentiated cells will eventually be lost from the system, unless these changes themselves confer self-renewing capabilities to cells. Here transit-amplifying cells are defined as those cells that possess the ability to undergo a limited number of cell replication events before undergoing apoptosis or differentiating further; unlike self-renewing cells, these cells cannot persist indefinitely in a tissue. Therefore, in tumors that are replenished by a small population of cancer stem cells, we consider that heterogeneity of transit-amplifying cells is proportional to the heterogeneity of cancer stem cells. Mutations conferring self-renewal propensities to transit-amplifying cells are not considered to contribute to the total (long-term) heterogeneity in this case. In tumor types that do not follow the cancer stem cell model, all cell types may accumulate alterations that have the potential to persist in the population, and therefore all tumor cells are included in the model describing tumors of this latter type. Mathematical models describing more complex population structures will be the topic of future investigations.

### Computer simulations

We perform exact computer simulations of the stochastic process; see the supplement online for the source code of the simulations. There are two categories of cell types: the cell types that harbor only neutral alterations (type A cells), and the cell types that additionally carry an advantageous mutation (type B cells). Denote their respective abundances by 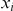 and 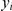, where 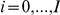 enumerate the individual cell types. The quantity 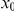 denotes the number of type A cells that carry no mutations, while 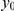 denotes the number of type B cells that harbor no neutral mutations. The index 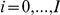 enumerates the individual cell types, where *I* denotes the maximum number of types for type A and B cells. This maximum number may be dictated by the number of ways in which genetically distinct cells can arise, or alternatively by the number of ways in which currently available genome profiling methods can distinguish differences between cells; in the latter case, differences are only noted when they occur in those parts of the genome which are profiled in a given study, and may underestimate the true extent of diversity in a tumor sample.

Initially, there are *N* unmutated cells, 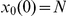, while 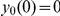 and 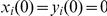 for 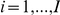. At each time step of this stochastic process, one cell is chosen for reproduction at random, but proportional to fitness. If there are *j* type B cells with fitness *R*+*S* in a population of *N*-*j* type A cells with fitness *R*, then the probability that a type B cell is chosen for reproduction is 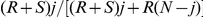. The chosen cell produces a daughter cell, possibly with a mutation, which replaces another randomly chosen cell. The total number of cells remains strictly constant. This stochastic process is known as the Moran model [28]. Each time a mutational event occurs, a new cell type is created; for instance, a cell of type 3 may produce a cell of type 9, if there are already 8 cell types present. For each parameter set, we perform many independent runs of the stochastic process to account for random fluctuations, and determine the heterogeneity of cells as
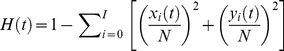
(1)for each time *t* (Fig. 1). This quantity is known as Simpson's index [31].

### The dynamics of heterogeneity due to neutral variation

Let us first investigate the level of heterogeneity of a population of type A tumor cells with growth rate *R* and death rate *D*, in which neutral alterations arise with probability *u* per cell division. We will consider advantageous mutants in a later section. During a small time interval of length 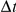, a cell division, death, or mutation event may occur. Starting from *N* cells at time *t*, there are 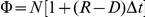 cells at time 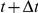: the number of cells that remain unchanged during 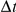 is given by 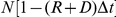, the number of cells that die during 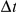 is given by 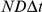, the number of cells that divide without mutating is given by 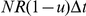 (producing 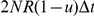 cells at time 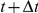), and the number of cells that divide while mutating is given by 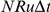 (producing 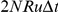 cells at time 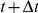, half of which carry the new mutation). Then the total number of cells at time 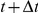 except novel mutants is given by 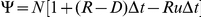. The probability that a randomly chosen pair of cells has exactly the same genotype is given by
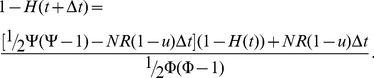
(2)


The factor 1/2 arises since each pair of cells is counted only once. When neglecting terms of higher order of 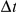, we obtain
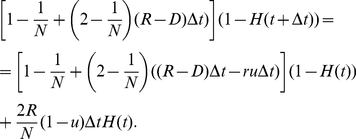
(3)


Then, letting 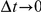 and with 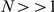 and 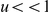, we have
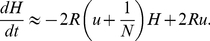
(4)


With 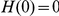, we obtain
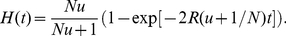
(5)


**Figure 2:**
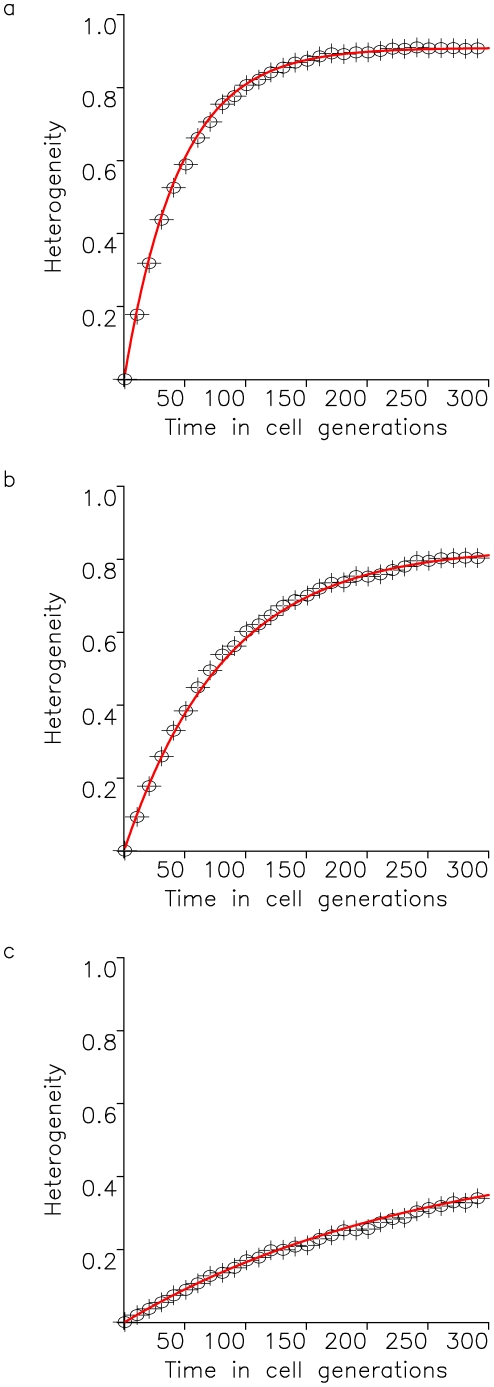
Tumor heterogeneity due to neutral variation. A cell population of fixed size, *N*, accumulates neutral mutations at rate *u* per cell division. All cell types have growth rate *R* and death rate *D*. The figure shows the fit of the exact stochastic computer simulation of this process with equation (5). In the simulation, heterogeneity is defined as Simpson's index, equation (1). Parameters are 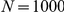, 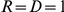, 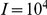, and 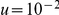 in (a), 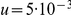 in (b), and 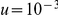 in (c), and results are averaged over 100 simulation runs.

This quantity increases smoothly from 0 to the asymptotic level, 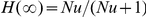. Figure 2 shows the dynamics of heterogeneity as given by equation (5) and the exact computer simulation of the stochastic process.

### The dynamics of heterogeneity due to neutral and advantageous variation

Let us next discuss the situation in which a single type B cell carrying an advantageous mutation arises at time 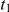. This cell has division and death rates of *R*+*S* and *D*, respectively, and its lineage accumulates neutral variation at rate *u* per cell division. Denote the number of type B cells at time *t* by 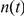 while the total population size is *N*. Type B cells have a per capita division rate of 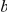, which includes the fitness values of both type A and B cells since it is the outcome of their competition. The probability of cell division of type B cells in a time interval of length 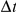 is given by
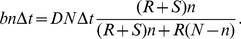
(6)


Then the per capita net growth rate of type B cells is given by 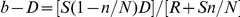 and the population of those cells grows according to
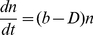
(7)with initial condition 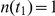.

To derive the expression for heterogeneity among type B cells over time, recall equation (5). With initial condition 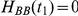 and given equation (7), the heterogeneity among type B cells is then given by
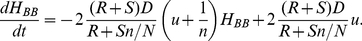
(8)


Similarly, the per capita cell division rate of type A cells is given by 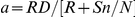. Then the heterogeneity among type A cells over time, 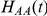, is given by
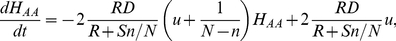
(9)where 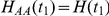 from equation (5). Finally, the total heterogeneity is composed of heterogeneity among type A cells, 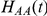, heterogeneity among type B cells, 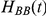, and heterogeneity between type A and B cells, 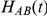. If the type B lineage does not have the possibility of going extinct, then the total heterogeneity of the population is given by
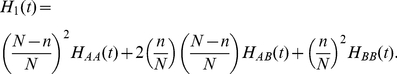
(10)


Considering the possibility of extinction of type B cells, the total heterogeneity becomes



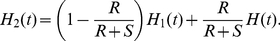
(11).

**Figure 3 pone-0017866-g003:**
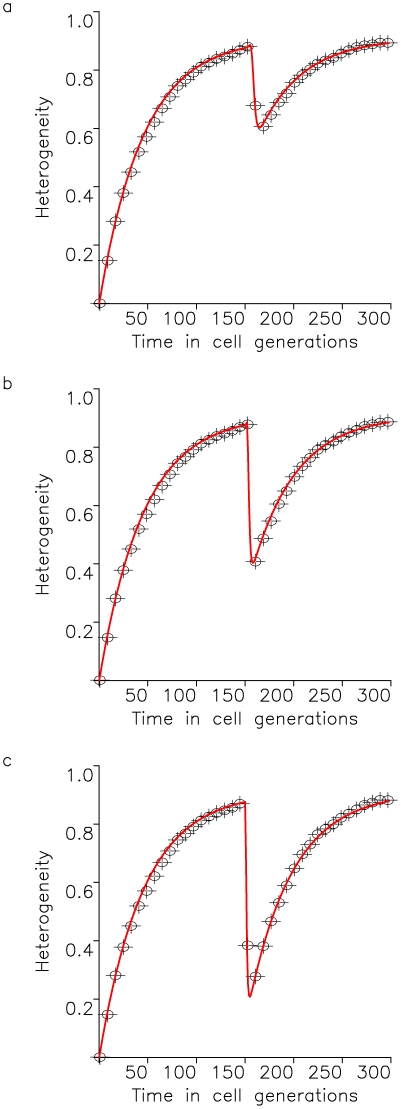
Tumor heterogeneity due to neutral and positively selected variation. A cell population of fixed size, *N*, accumulates neutral mutations at rate *u* per cell division. All neutral mutants have growth rate *R* and death rate *D*. Additionally, a positively selected mutant arises at time 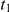 in the population. Its lineage has growth rate 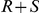 and death rate *D*. The figure shows the fit of the exact stochastic computer simulation of this process with equation (11). In the simulation, heterogeneity is defined as Simpson's index, equation (1). Parameters are 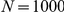, 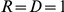, 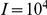, 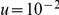, and 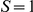 in (a), 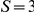 in (b), and 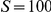 in (c), and results are averaged over 100 simulation runs.

Here the factor 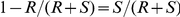 denotes the probability that a newly arisen type B cell survives the stochasticity when its clone is small; this calculation is based on the branching process approximation (e.g., [32]). Since cell types A and B differ by at least the advantageous mutation, we can consider 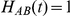 for all times *t*. Figure 3 shows the dynamics of heterogeneity as given by equation (11) and the results of the exact stochastic computer simulation.

**Figure 4 pone-0017866-g004:**
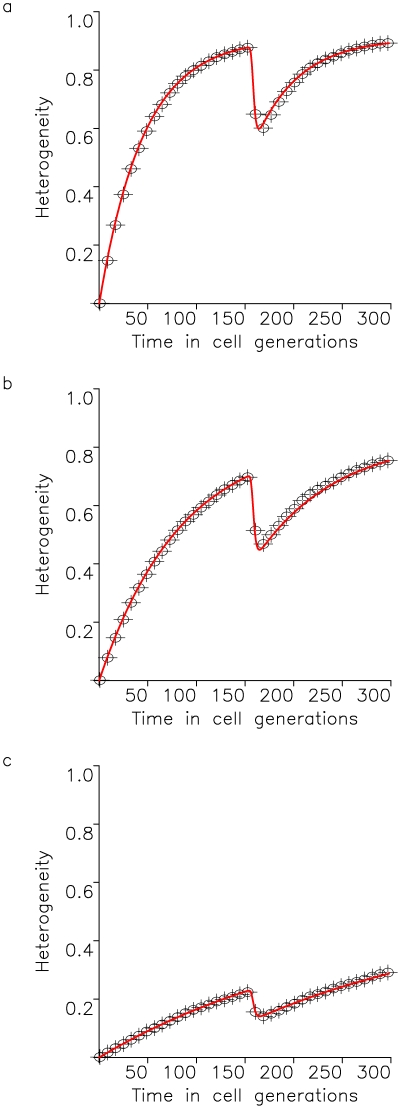
Tumor heterogeneity when only neutral variation can be detected. A cell population of fixed size, *N*, accumulates neutral mutations at rate *u* per cell division. All neutral mutants have growth rate *R* and death rate *D*. Additionally, a positively selected mutant arises at time 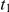 in the population. Its lineage has growth rate 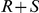 and death rate *D*. This mutation, however, cannot be detected when measuring heterogeneity in the population. The figure shows the fit of the exact stochastic computer simulation of this process with equation (12). In the simulation, heterogeneity is defined as Simpson's index, equation (1), and wild type cells as well as cells harboring the advantageous mutation are considered as one cell type. Parameters are 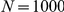, 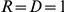, 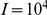, 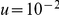, and 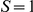 in (a), 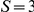 in (b), and 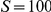 in (c), and results are averaged over 1000 simulation runs.

Alternatively, consider the case in which the advantageous mutation cannot be detected by currently used screening methods. In that case, the only loci contributing to heterogeneity are those that do not cause a fitness difference when mutated – i.e., neutral variation. Then the heterogeneity between type A and B cells at time *t*, 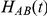, is determined by considering the ancestral cell lineages giving rise to any pair of cells. Recall that the number of type B cells existing at time 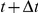 is 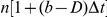, and the number of cells harboring a novel neutral mutation at time 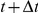 is 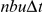. Then the probability that a cell accumulates a mutation between times *t* and 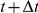 is given by 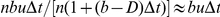. As we trace the ancestral lineage of a type B cell from time *t* back to time 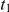, the probability that no mutation occurs during this time interval is given by 
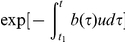
. Similarly, the probability that no mutation occurs in the ancestral lineage of a type A cell is given by 
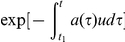
, where *a* denotes the per capita division rate of type A cells. Then we have
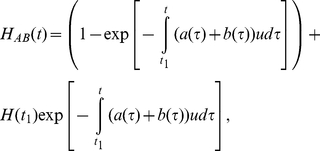
(12)since a pair of type A and B cells are different either because their ancestral cells at time 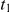 were different, or because the ancestral lineage of either cell accumulated a mutation. Equation (12) can be approximated by
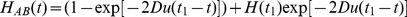
(13)since 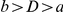. Figure 4 shows the dynamics of heterogeneity in the case in which the advantageous mutation cannot be detected, as given by equation (12), and the results of the exact stochastic computer simulation for that scenario.

Finally, let us discuss the situation in which type B cells carrying advantageous mutations arise at rate *v* per cell division. A cell with *k* advantageous mutations has growth and death rates of 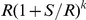 and *D*, respectively, and its lineage accumulates neutral variation at rate *u* per cell division. In this case, the rate at which a selective sweep occurs is given by 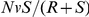. Then the expected mean heterozygosity is given by
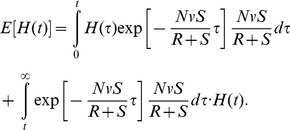
(14)


**Figure 5:**
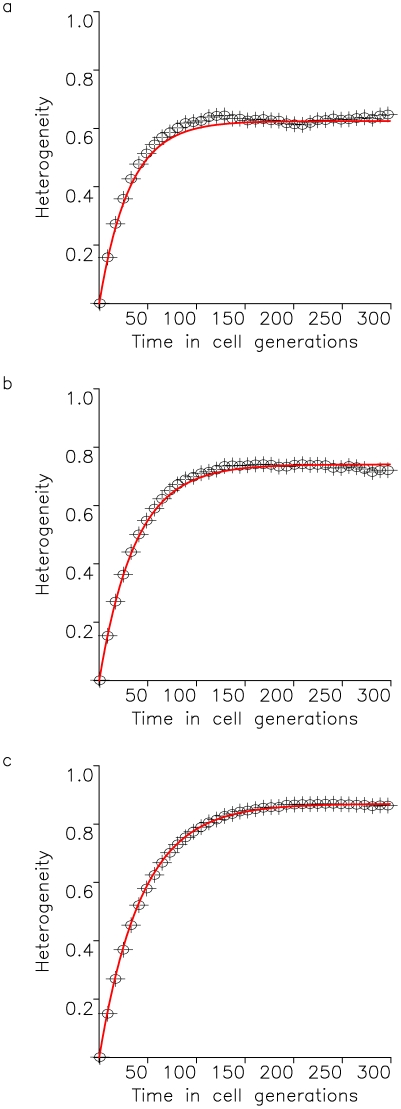
Tumor heterogeneity due to continuously arising neutral and advantageous variation. A cell population of fixed size, *N*, accumulates neutral mutations at rate *u* per cell division and advantageous mutations at rate *v* per cell division. All neutral mutants have growth rate *R* and death rate *D*, while a cell with *k* advantageous mutations has growth and death rates of 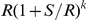 and *D*. The figure shows the fit of the exact stochastic computer simulation of this process with equation (15). In the simulation, heterogeneity is defined as Simpson's index, equation (1). Parameters are 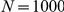, 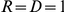, 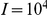, 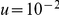, 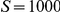, and 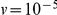 in (a), 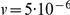 in (b), and 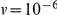 in (c), and results are averaged over 1000 simulation runs.

In this equation, the first term corresponds to the case in which one or more selective sweeps of advantageous mutants occur before time *t*, while the second term corresponds to the case in which no selective sweep has occurred until time *t*. By substituting equation (1) for *H*(*t*), we obtain
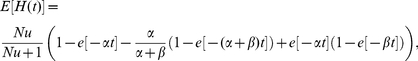
(15)where 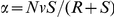 and 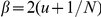. In the limit of infinitely large time *t*, this expression becomes 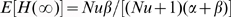. Figure 5 shows the dynamics of heterogeneity in the case in which advantageous mutations arise at rate *v* while neutral mutations are accumulated at rate *u* per cell division; we show equation (15) and the results of the exact stochastic computer simulation for that scenario.

So far, we have discussed the dynamics of intratumor heterogeneity in populations of constant size. However, since tumors expand over time, it is also important to consider the accumulation of variation in growing tumor cell populations. In cases in which the expansion of a tumor cell population is driven by the accumulation of (epi)genetic alterations, the model described above can be used to describe the kinetics of diversity during those times when the population size is roughly constant – i.e., between the events of accumulation of a novel advantageous mutation. Once such a mutation has arisen, the population grows until reaching a new steady state level. Our model is useful for describing the accumulation of variation during these fixed-size periods. Alternatively, an exponentially growing population can be considered in which alterations arise; a useful stochastic process for this scenario is the branching process model in which cells divide in a binary fashion, and the population grows (or declines) on average exponentially. Such a model is the topic of other work [33], [34].

## Discussion

Intratumor heterogeneity is a key mechanism underlying tumor progression and the frequent lack of therapeutic responses. Although tumors are thought to originate from a single cell that accumulates (epi)genetic alterations necessary for transformation, by the time of diagnosis, most cancers exhibit widespread heterogeneity. This tumor diversity does not only complicate the profiling of cancers, since a sample may not be representative of the whole, but also decreases the likelihood of cure due to therapeutic interventions since resistant clones may already pre-exist therapy. An understanding of the evolutionary forces driving intratumor heterogeneity would enhance our understanding of tumorigenesis and may allow us to more effectively plan treatments.

In this paper, we have designed a stochastic mathematical model of the accumulation of (epi)genetic alterations in populations of cells to estimate the extent of heterogeneity over time (Fig. 1). We have studied this model with exact stochastic computer simulations (Figs. 2–5) and have also derived analytical approximations for the dynamics of heterogeneity in the population. We have considered two different types of heritable alterations that may arise during cell divisions: neutral variation that does not change the fitness of cells, but leads to the emergence of a new cell type that can be distinguished from the resident cancer cell population with molecular profiling techniques, and advantageous alterations which lead to a fitness increase of the cell and potentially a selective sweep in the population. Here selective sweep refers to the reduction or elimination of variation in a cell population due to recent and strong positive selection [35]. We have neglected disadvantageous alterations since in large tumors, cells carrying such alterations are likely unable to establish a surviving clone, but go extinct due to their deleterious characteristics. The population of cells at risk of accumulating these alterations consists of those cells that are maintained in the population for long time horizons; in tumor types adhering to the cancer stem cell hypothesis, only cancer stem cells have self-renewal propensities and hence, genetic variability accumulated within them may persist in the population rather than being lost due to differentiation and death of its carrier cell. Heterogeneity accumulated in progenitors and differentiated cells can contribute to a snapshot analysis of diversity, but cannot be maintained for long time horizons. In the case of tumors not following the cancer stem cell model, all tumor cells potentially possess self-renewal abilities and are therefore part of the population of cells which accumulate (epi)genetic variability.

Our evolutionary model demonstrates that the accumulation of neutral variation in a population leads to an increase in heterogeneity until a maximum extent is reached (Fig. 2). This maximum value is dictated by the number of cells in the population as well as the mutation rate giving rise to new cell types. After introduction of an advantageous mutation, the extent of heterogeneity decreases rapidly as the advantageous clone spreads through the population, but afterwards rebounds as neutral variation continues to be accumulated in this clone (Figs. 3 and 4). If successively more advantageous mutants emerge in the population over time, then the extent of heterogeneity is maintained at a lower level since the advantageous clones arise stochastically and decrease the average heterogeneity across the cancer cell population (Fig. 5). Our model provides an understanding of the consequences of accumulating neutral and advantageous variation in a tumor; such knowledge aids in the interpretation of cancer genomic data as well as relays an understanding of the basic biology and kinetics of tumors.

We have chosen to concentrate on the behavior of tumor cells which accumulate (epi)genetic changes while proliferating in a fixed-size population or niche. Situations with exponentially increasing population sizes are the topic of follow-up work [33], [34]. For clarity, we have neglected other important aspects contributing to tumorigenesis, such as interactions of tumor cells with the immune system and microenvironment, cell-cell interactions, competition for resources and space, as well as the effects of exogenous mutagenic factors and inherited predispositions. These factors will be considered in future contributions.

To validate the predictions of this model and further the understanding of tumor diversity, detailed experimental analyses of tumor heterogeneity are necessary. For instance, the number and frequencies of (epi)genetically or morphologically diverse clones in a tumor should be ascertained along with the variability of these quantities when comparing tumors from different patients or different sites within the same patient, such as primary and metastatic lesions. With such data, it will be possible to relate the extent of diversity to clinically important covariates like survival, proliferation indices, invasiveness, sensitivity to therapy, etc. Such studies have recently been initiated [36]–[38] and will enhance our understanding of the role of tumor diversity in cancer progression and the response to treatment. Furthermore, evolutionary parameters such as growth and death rates, mutation rates, and the ability of different cell types to migrate, adhere and invade are needed to accurately describe the dynamics of diversity. The values of such parameters remain unknown for many cell and mutation types, but are necessary for progress in this field. Finally, the role of the immune system and microenvironment in tumor progression and diversity must be delineated to design accurate mathematical models. Although such studies have been initiated [39], [40], many open questions remain. In summary, experimental methodologies to profile single cells – both tumor and microenvironmental – from neoplasms at multiple stages of their evolution are necessary such that, with the help of appropriate analysis tools, the clinical management of patients with premalignant lesions or cancer can be improved.
